# Refractive outcome and lens power calculation after intrascleral intraocular lens fixation: a comparison of three-piece and one-piece intrascleral fixation technique

**DOI:** 10.1186/s40662-023-00341-6

**Published:** 2023-06-09

**Authors:** Markus Schranz, Adrian Reumüller, Klaudia Kostolna, Caroline Novotny, Daniel Schartmüller, Claudette Abela-Formanek

**Affiliations:** Department of Ophthalmology and Optometry, Medical University of Vienna, Währinger Gürtel 18-20, 1090 Vienna, Austria

**Keywords:** Secondary IOL, Refractive prediction error, IOL subluxation, Scleral IOL fixation, IOL power formulae, Secondary IOL tilt, Secondary IOL decentration

## Abstract

**Purpose:**

To evaluate the refractive prediction error of common intraocular lens (IOL) power calculation formulae in patients who underwent intrascleral IOL fixation using two different techniques.

**Methods:**

This is a prospective, randomized, longitudinal, single-site, single-surgeon study. Patients who underwent intrascleral IOL implantation using the Yamane or the Carlevale technique were followed up for a period of six months postoperatively. Refraction was measured using the best-corrected visual acuity at 4 m (EDTRS chart). Lens decentration, tilt and effective lens position (ELP) were assessed using an anterior segment optical coherence tomography (AS-OCT). The prediction error (PE) and the absolute error (AE) were evaluated for the SRK/T, Hollayday1 and Hoffer Q formula. Subsequently, correlations between the PE and axial length, keratometry, white to white and ELP were assessed.

**Results:**

In total, 53 eyes of 53 patients were included in the study. Twenty-four eyes of 24 patients were in the Yamane group (YG) and 29 eyes of 29 patients were in the Carlevale group (CG). In the YG, the Holladay 1 and Hoffer Q formulae resulted in a hyperopic PE (0.02 ± 0.56 D, and 0.13 ± 0.64 D, respectively) while in the SRK/T formula the PE was slightly myopic (− 0.16 ± 0.56 D). In the CG, SRK/T and Holladay 1 formulae led to a myopic PE (− 0.1 ± 0.80 D and − 0.04 ± 0.74 D, respectively), Hoffer Q to a hyperopic PE (0.04 ± 0.75 D). There was no difference between the PE of the same formulae across both groups (*P* > 0.05). In both groups the AE differed significantly from zero in each evaluated formula. The AE error was within ± 0.50 D in 45%–71% and was within ± 1.00 D in 72%–92% of eyes depending on the formula and surgical method used. No significant differences were found between formulae within and across groups (*P* > 0.05). Intraocular lens tilt was lower in the CG (6.45 ± 2.03°) compared to the YG (7.67 ± 3.70°) (*P* < 0.001). Lens decentration was higher in the YG (0.57 ± 0.37 mm) than in the CG (0.38 ± 0.21 mm), though the difference was not statistically significant (*P* = 0.9996).

**Conclusions:**

Refractive predictability was similar in both groups. IOL tilt was better in the CG, however this did not influence the refractive predictability. Though not significant, Holladay 1 formula seemed to be more probable than the SRK/T and Hoffer Q formulae. However, significant outliers were observed in all three different formulae and therefore remain a challenging task in secondary fixated IOLs.

## Background

Clear lens exchange, early cataract surgery and the presence of pseudoexfoliation syndrome in combination with an increased life expectancy have led to an increase in subluxated and opacified intraocular lenses (IOLs) in recent years. There are several options for successful secondary lens implantation with scleral fixation of a posterior IOL [1, 2].

Scharioth and Pavlidis were the first to describe the intrascleral fixation of a three-piece IOL. In this technique, the haptics of the IOL are externalized through a 23G sclerotomy and placed in a 3 mm long, limbus parallel intrascleral tunnel, 180° apart. The immediate implantation of the haptic in the intrascleral tunnel can be challenging for the surgeon [3, 4]. To surpass this problem, Agarwal et al. placed the haptic below scleral flaps and introduced the tip of the haptic in a short tunnel. The flaps are subsequently glued using fibrin tissue glue [5, 6]. Posterino et al. described a technique in which scleral pockets are used to store the C-loop haptics [7].

The transconjunctival, intrascleral double-needle flanged IOL fixation technique of a three-piece IOL described by Yamane has become a popular and frequently implemented method in the absence of capsular support [8]. Precise and symmetrical externalization and flanging of the haptics is paramount to reduce IOL decentration and tilt.

A recent method is the four-flanged technique first described by Canabrava. In this technique, a four-loop haptic IOL is fixated to the scleral using Prolene sutures with flanged ends [9]. These techniques are all based on the off-label use of an IOL designed for in-the-bag implantation.

In 2015, a new IOL especially designed for sutureless scleral fixation, the FIL-SSF Carlevale lens (Soleko S.p.A., Rome, Italy), was introduced [10]. In contrast to the Yamane IOL implantation technique, the single-piece Carlevale IOL is made of hydrophilic acrylic and has two anchor haptics designed for intrascleral placement allowing for a standardized and reproducible surgical procedure [11, 12]. The haptics of the Carlevale IOL are placed between 1.5 and 2.5 mm behind the limbus irrespective of axial length and anatomy of the anterior segment.

Besides advantages and disadvantages between the surgical techniques, such as the necessity of peritomies and the use of intraocular forceps or needles and thermocauterization, the immediate availability of the IOLs is an important factor in the clinical routine. Three-piece IOLs are commonly available in surgery rooms as backup lenses for sulcus IOL implantation, whereas the FIL-SSF Carlevale IOL often has to be ordered or is not readily available.

The clinical outcome of these methods was the topic of numerous papers in recent years. However, little has been reported regarding refractive outcome, an important issue in an era of increasing patients refractive expectation [3].

Different lens designs, fixation methods and location of haptic externalization from 1.5 to 2.5 mm posterior to the limbus complicate the prediction accuracy of the refractive outcome in scleral fixated lenses and make it much more unpredictable as compared to an in-the-bag IOL implantation. To the best of our knowledge, this is the first study comparing the refractive outcome and prediction error (PE) in a three-piece and one-piece intrascleral fixation technique.

## Methods

This prospective, longitudinal, single-site, single-surgeon study was conducted at the Medical University of Vienna. All study procedures adhered to the Tenets of the Declaration of Helsinki. The study was approved by the local ethics committee of the Medical University of Vienna (EK 2301/2019). All patients provided written informed consent prior to inclusion to the study.

## Inclusion and exclusion criteria

Patients in need of secondary IOL implantation due to spontaneous late in-the-bag IOL dislocation, aphakia after complicated cataract surgery, blunt non-penetrating and perforating trauma or IOL opacification were included between February 2020 and July 2021 and randomized to either the Yamane group (YG) receiving an Aspira 3PaVa IOL (Human Optics Holding AG, Erlangen, Germany) or the Carlevale group (CG) receiving the FIL-SSF IOL (Soleko, Rome, Italy).

Exclusion criteria were history of buckling surgery, corneal ectasia, corneal degeneration, corneal dystrophy, status after corneal surgery, open globe trauma, amblyopia, advanced macular disease with impact on visual function, or postoperative visual acuity lower than 20/63 (Snellen).

### Examinations

Main outcome measure was postoperative subjective refraction using EDTRS charts at a distance of 4 m. Secondary outcomes included IOL position, decentration and tilt.

Biometric parameters such as axial length (AL), keratometry (Kmean of anterior Kvalues), and white-to-white distance (WTW) were measured at baseline with the IOLMaster 700 (Carl Zeiss Meditec Inc., Dublin, CA, USA) prior to pupil dilation. Biometry was feasible in all study eyes. Best corrected visual acuity testing was performed 6 months (24 ± 2 weeks) postoperatively using EDTRS charts at 4 m as well as IOL position, decentration and tilt measurement with an anterior segment optical coherence tomography (AS-OCT) (Casia 2-Tomey, Japan).

**Fig. 1:**
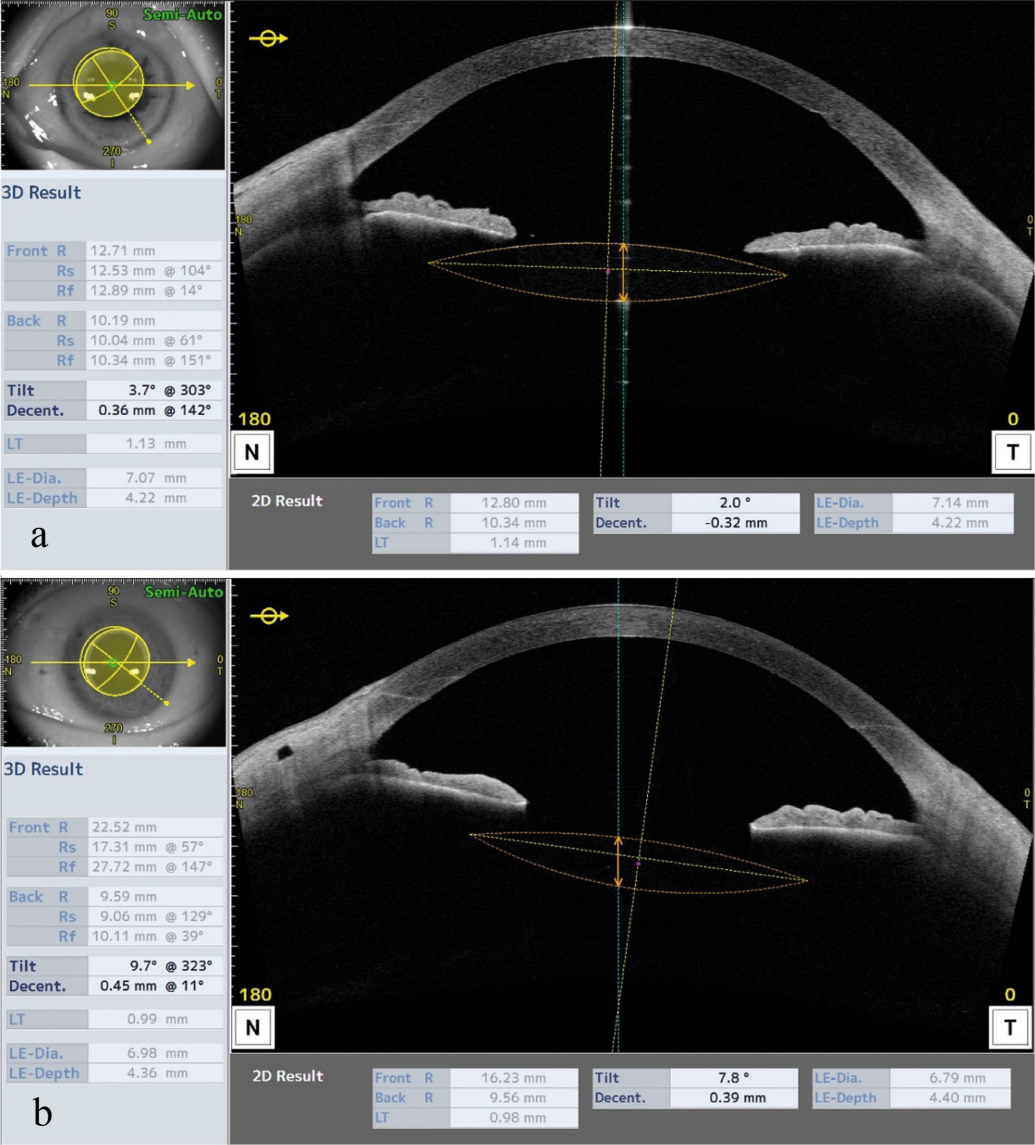
Anterior segment optical coherence tomography images of the Yamane fixated three-piece intraocular lens (IOL) (**a**), and the Carlevale FIL SFF6 IOL (**b**)

Decentration and tilt were measured using the inbuilt software of the Casia 2 AS-OCT analyzing and modelling the IOL position over 360 degrees (see Fig. 1). Effective lens position (ELP) was defined as the length of a perpendicular line between the endothelial vertex of the cornea and the anterior surface of the IOL.

#### Surgical techniques

All surgeries were performed by a highly experienced surgeon (CAF) with patients under general or retrobulbar anesthesia.

#### Yamane technique

Three 23-gauge (G) vitrectomy ports were placed 3.5 mm posterior to the limbus for 23-G vitrectomy. At 90°, a 2.7 to 3.0 mm posterior limbal incision (PLI) was made. Two paracenteses were performed. If an IOL was preexistent, it was maneuvered into the anterior chamber, cut in half, and removed. 360° vitrectomy was performed using the Oertli OS4 system (Oertli Instrumente AG, Berneck Switzerland).

**Fig. 2:**
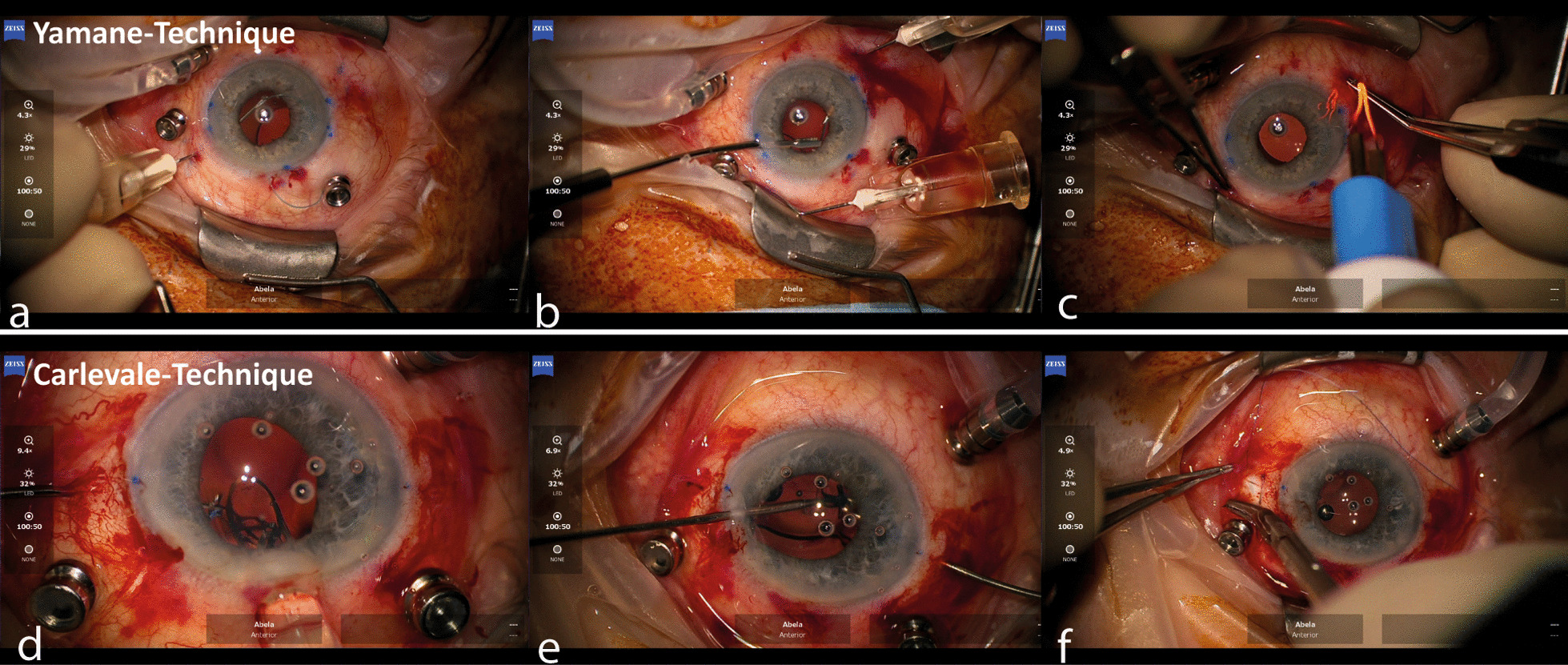
Image captures of the two surgeries. Images (**a**–**c)** show the Yamane technique. The leading haptic of the three-piece intraocular lens (IOL) was inserted in the 30 gauge hypodermic needle (**a**). The trailing haptic was inserted thereafter (**b**). After externalizing both haptics 2.5 mm posterior to the surgical limbus, both haptic ends were flanged (**c**) and inserted into the scleral tunnels. Images (**d**–**f**) show the Carlevale-technique. The leading haptic was externalized through the sclerotomy 1.5 mm posterior to the surgical limbus using a 24 gauge intraocular forceps (**d**). Subsequently, the leading haptic was externalized using a handshake technique through the opposite sclerotomy (**e**). The T-haptics were then placed into the scleral pockets, which were then closed using two single knot 8.0 polyglactin sutures on each side (**f**)

Subsequently, the two haptic externalization points were marked 2.5 mm posterior to the surgical limbus (blue line) at 30° and 210° using a surgical marker. The Aspira® 3PaVA, three-piece IOL was then placed in the anterior chamber using the Unfolder Emerald XL handpiece and cartridge implantation series while the trailing haptic was left in the posterior limbal incision. A micro forceps was used to feed the leading haptic into a 30-G bent needle (see Fig. 2), which penetrated the sclera anti-clockwise through a 2 mm limbus parallel long intrascleral tunnel. The same procedure was then performed with the trailing haptic while the first needle remained in place, still holding the leading haptic. In the next step, both needles were simultaneously removed leading to a smooth externalization of the leading and trailing haptics. The IOL optic was then centered by gentle manipulation of the externalized haptics. The haptic ends were shortened if necessary and flanged using a single use electrocautery and then stored in the intrascleral tunnel. Finally, the viscoelastic was removed from the vitreous cavity and the anterior chamber. Pars plana ports were removed and the corresponding sclerotomies sutured using 8.0 polyglactin sutures, the corneal incisions were sealed using basic salt solution. 0.1mL Cefuroxime was injected in the anterior chamber at the end of surgery.

#### Carlevale technique

In the Carlevale group, posterior limbal incision, paracenteses, vitrectomy and lens removal were analogously performed to the YG. After marking the 0° axis on the limbus using a Mendez Ring and a surgical marker, the conjunctiva was opened at opposite sides of the cornea around this axis. Two 2.5 mm long, 300 μm deep scleral incision using a diamond blade radial to the cornea and posterior to the limbus were performed at 0° and 180°. Perpendicular to these incisions, two opposite facing 2.5 mm deep scleral pockets, on the nasal and temporal sides, were created using a micro-crescent blade. A 23-G lancet was used to perform a sclerotomy 1.5 mm posterior to the surgical limbus (blue line) within the radial scleral incision.

The Carlevale SSF-IOL was ejected and implanted into the anterior chamber (see Fig. 2). During implantation of the IOL, the leading T-haptic was grasped with a 24-G forceps (MST micro holding forceps) through the left sclerotomy and externalized. The T-haptic unfolds and functions as an anchor. The trailing haptic was then grasped through the opposite paracentesis and passed to the 24-G forceps coming through the right sclerotomy in a handshake technique. The haptic was then externalized in the manner of the leading one. Afterwards, both ends of the T-haptics were placed in the scleral pockets which were then adapted and sutured with 2 limbus parallel 8.0 polyglactin. The surgery was completed analogously to the YG.

### Data analysis and statistical testing

Anterior chamber depth (ACD) independent formulae only were used for IOL power calculation. Those were the SRK/T, Holladay 1 and Hoffer Q, the corresponding constants are shown in Table 3. For the Aspira 3PaVA the constants given by the company were used. In contrast, for the FIL-SSF IOL, the constants suggested by Vaiano et al. were used [11]. For calculation of the PE, we subtracted the predicted refractive outcome, corresponding to the IOL power and formula, from the measured postoperative spherical equivalent (SE).

A negative PE indicated a myopic result whereas a positive PE indicated hyperopic result as compared to the expected refraction. Subsequently, we calculated the arithmetic mean for each formula and lens type. For comparing the PE of different formulae within groups we used two-way ANOVA, Bonferroni correction was used to correct for multiple testing. Differences in the PE across groups were compared using two-way ANOVA, Sidak’s test was used for multiple comparison. The absolute prediction error (AE) was defined as the absolute value of the PE of each patient, subsequently the mean AE (MAE) and the median AE (MedAE) were calculated.

Differences in the AE between formulae within groups were calculated using Friedman’s test and differences between formulae across groups were calculated using Kruskal-Wallis test. Dunn’s test was used for multiple comparison. Univariable and multivariable linear regression analyses were used for analyses of correlations between PE and biometry data (AL, ELP, Kmean), lens decentration and tilt.

Evaluation for normal distribution was done using boxplots and the Shapiro-Wilk test. Normal distributed data was reported as mean ± standard deviation and Student t-test or paired Student t-test were used to compare the means between independent or dependent groups, respectively.

Non-normally distributed data was reported as median and interquartile range (1st quartile; 3rd quartile). The level of significance was set to α = 0.05. For statistical testing, SPSS (Version 25.0; Armonk, NY: IBM Corp.) and GraphPad Prism 9 (Version 9.3.0 (345), November 11, 2021, GraphPad Software, LLC) were used.

## Results

In total, 57 patients were screened, two did not meet inclusion criteria due to keratoconus and perforating keratoplasty, another two had to be excluded due to cystoid macular edema at six months (both in the YG). In total, 53 eyes of 53 consecutive patients fit the inclusion criteria for this analysis. Indications for surgery can be seen in Table 1. Patient demographics are listed in Table 2. Refractive outcome PE, MAE, MedAE, for the SRK/T, Holladay 1 and the Hoffer Q formula are reported in Table 3.

**Table 1:** Indications for surgery

Parameters	Number of eyes		
	Total	Yamane group	Carlevale group
Pseudoexfoliation syndrome	18	8	10
Complicated cataract surgery	8	5	3
IOL dislocation due to high myopia	4	1	3
Non penetrating blunt trauma	4	3	1
Vitrectomy	4	1	3
IOL opacification	4	2	2
Ectopia lentis due to Marfan syndrome	1	1	0
Spontaneous in the bag dislocation (idiopathic)	10	3	7
Total	53	24	29

**Table 2:** Patient demographics

Parameters	Yamane	Carlevale	*P* value
Age (years)	68.5 ± 14.7	71.1 ± 11.9	0.489
Gender (male/female)	13/11	15/14	
Spherical equivalent (D)	− 0.79 ± 1.07	− 0.70±1.30	0.999
Visual acuity (logMAR)	0.18 ± 0.51	0.13 ± 0.57	0.773
Axial length (mm) (Range)	24.36 ± 1.52 (22.10 to 27.92)	24.59±1.90 (21.44 to 29.41)	0.999
Kmean (D)	7.90 ± 0.36	7.77 ± 0.25	0.999
White to white (mm)	11.92 ± 0.46 (11.10 to 13.20)	12.15±0.35 (11.40 to 12.70)	0.999
Effective lens position (mm)	4.05 ± 0.38	4.55±0.44	0.727
Tilt (degree)	7.67 ± 3.70 (2.5 to 17.4)	6.45 ± 2.03 (3.4 to 13.3)	0.004*
Decentration (mm)	0.57 ± 0.37	0.38 ± 0.21	0.999

*D* = diopters; *mm* = millimeters; *Kmean* = mean keratometry

* Significant difference between both groups

Six months postoperatively, when visual acuity testing and AS-OCT imaging is performed, neither of the patients presented with retrograde iris block, optic-iris-capture nor scleral thinning, haptic extrusion or intrusion. However, two patients in the CG presented with mild self-limiting postoperative vitreous hemorrhage.

**Table 3:** Refractive outcomes

Formula		Constant	APE (D)	MAE (D)	MedAE (D)	% within ±0.5D	% within ± 1.0 D	% within ± 2.0 D
SRK/T	Yamane	119.10	− 0.16 ± 0.56	0.52	0.47	54.0	87.5	100
	Carlevale	118.92	− 0.10 ± 0.80	0.68	0.52	44.8	72.4	100
Holladay 1	Yamane	1.73	0.02 ± 0.56	0.45	0.38	62.5	91.7	100
	Carlevale	1.75	− 0.04 ± 0.74	0.61	0.49	51.7	75.9	100
Hoffer Q	Yamane	5.34	0.13 ± 0.64	0.45	0.29	70.8	83.3	100
	Carlevale	5.48	0.04 ± 0.75	0.62	0.60	44.8	75.9	100

*D* = diopters; *APE* = arithmetic prediction error; *MAE* = mean absolute prediction error; *MedAE* = median absolute prediction error; *% within* = the percentage of eyes within a certain prediction error

Comparing the PEs within the YG, we found that the SRK/T formula resulted in a myopic PE whereas Holladay 1 formula and HofferQ formula resulted in a hyperopic PE. The difference in the PE between the SRK/T and Holladay 1 formula (*P* = 0.0029) and the SRK/T and Hoffer Q formula (*P* = 0.0127), was significant, but no statistical difference was found between the Holladay 1 and Hoffer Q formula (*P* = 0.1585).In the CG, SRK/T and Holladay I resulted in a myopic PE whereas Hoffer Q resulted in a hyperopic PE. The difference in the PE between the SRK/T and Hoffer Q formula (*P* = 0.0161) was significant as was the Hoffer Q and Holladay 1 (*P* = 0.0347). No difference was found between Holladay 1 and SRK/T formula (*P* = 0.1795).

**Fig. 3:**
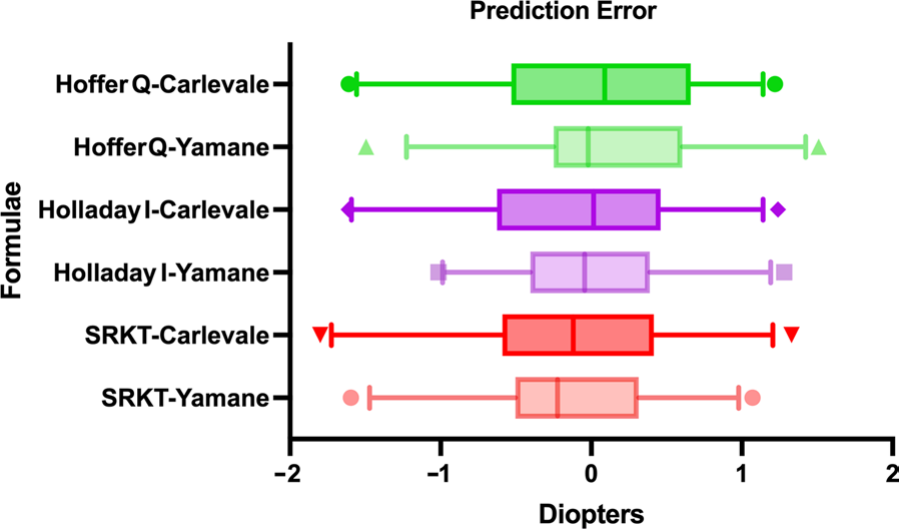
Box plot of the prediction error in both groups

Comparing the PE of corresponding formulae across groups, no significant differences were found for SRK/T (*P* = 0.9999), Holladay 1 (*P* = 0.9575) and Hoffer Q (*P* = 0.2118). Boxplots of the PEs are shown in Fig. 3.

**Fig. 4:**
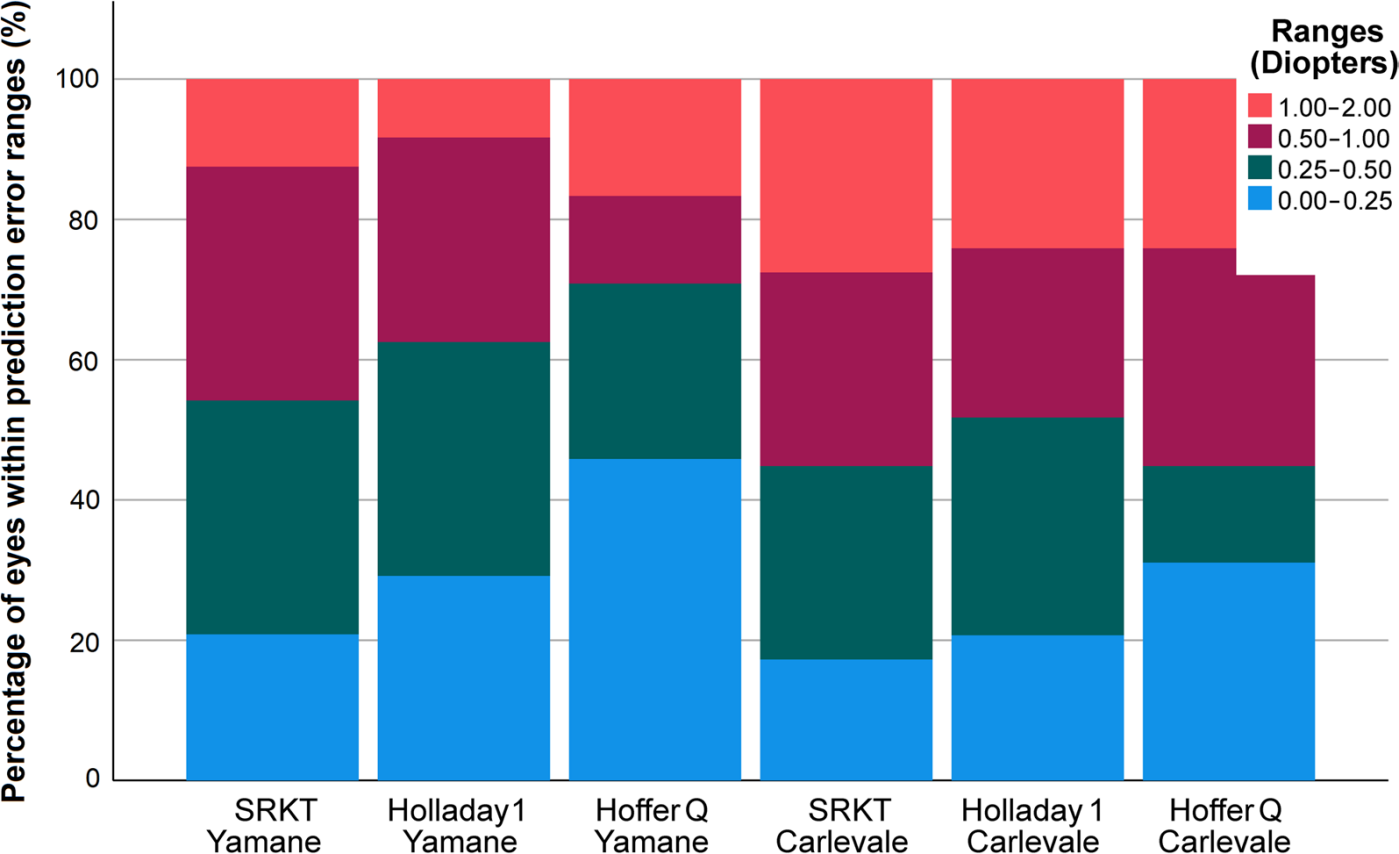
Stacked histogram comparing the percentages of eyes within a certain prediction error range

Each AE differed significantly from 0 D (SRK/T: *P* < 0.0001; Holladay 1: *P* < 0.0001, Hoffer Q: *P* < 0.0001). There was no significant difference within the AEs in the YG (*P* = 0.4169) or in the CG (*P* = 0.2785). No significant difference was found in the AE across groups (*P* = 0.2657). Stacked histogram of the percentages of eyes within certain error ranges can be seen in Fig. 4.

### Regression analyses

**Fig. 5:**
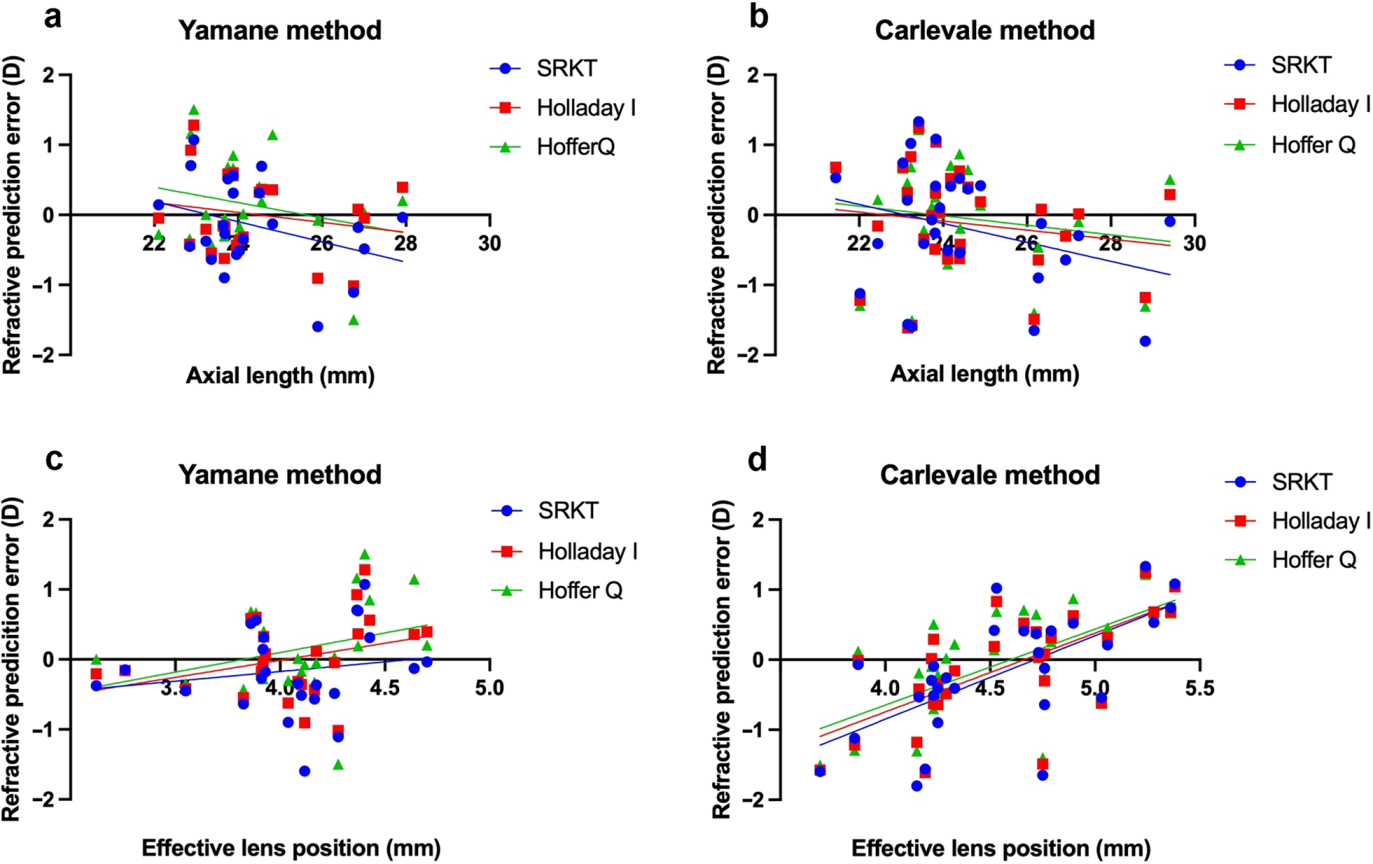
Scatterplots of the refractive prediction error vs. the effective lens position of the Yamane method (**a**) and the Carlevale method (**b**). Scatterplots of the refractive prediction error vs. the axial length of the Yamane method (**c**) and the Carlevale method (**d**). Corresponding estimates, R^2^ values and *P* values can be found in Table 4

In a univariable model, we calculated the correlation between the PE for each formula and the parameters AL, WTW, Kmean, ELP, decentration and tilt. In the YG, we found a significant correlation between the PE and the K mean value for the Hoffer Q formula (*P* = 0.03, R^2^ = 0.205). In the CG, we found significant correlations between the ELP and the PE of each formula (see Fig. 5). A detailed overview can be seen in Table 4.

**Table 4:** Correlation between APE and biometric parameters (univariable analysis)

Formula		Yamane				Carlevale			
		Intercept	Slope	Significancy of slope (*P* value)	R^2^	Intercept	Slope	Significancy of slope (*P* value)	R^2^
SRK/T	AL	3.36	− 0.14	0.106	0.120	3.13	− 0.14	0.108	0.093
	WTW	− 2.99	0.24	0.407	0.032	− 1.04	0.07	0.872	< 0.001
	Kmean	− 2.25	0.27	0.489	0.023	− 3.66	0.45	0.491	0.018
	ELP	− 1.28	0.28	0.432	0.028	−** 5.62**	**1.19**	**< 0.001**	**0.433**
	Decentration	− 0.16	0.01	0.976	< 0.001	0.04	− 0.56	0.468	0.021
	Tilt	− 0.48	0.04	0.231	0.065	0.23	− 0.06	0.445	0.023
Holladay 1	AL	1.74	− 0.07	0.390	0.040	1.46	− 0.06	0.421	0.024
	WTW	− 2.00	0.17	0.516	0.020	− 0.06	− 0.01	0.990	< 0.001
	Kmean	1.261	− 0.16	0.652	0.010	0.07	− 0.03	0.968	< 0.001
	ELP	− 1.96	0.49	0.118	0.107	−** 5.25**	**1.13**	**< 0.001**	**0.444**
	Decentration	0.10	− 0.16	0.634	0.011	0.14	− 0.63	0.388	0.029
	Tilt	− 0.27	0.04	0.244	0.061	0.50	− 0.09	0.217	0.058
Hoffer Q	AL	2.80	− 0.11	0.239	0.070	1.63	− 0.07	0.403	0.026
	WTW	− 0.89	0.09	0.773	0.004	− 0.69	0.05	0.897	< 0.001
	Kmean	6.58	− 0.82	0.030	0.205	1.12	− 0.15	0.810	0.002
	ELP	− 2.14	0.56	0.113	0.110	−** 4.89**	**1.08**	**< 0.001**	**0.397**
	Decentration	0.33	− 0.36	0.325	0.044	0.25	− 0.77	0.301	0.041
	Tilt	− 0.09	0.03	0.447	0.027	0.66	− 0.11	0.156	0.076

*APE* = arithmetic prediction error; *AL* = axial length; *WTW* = white to white; *Kmean* = mean keratometry; *ELP* = effective lens position

Table shows the correlation between the APE of the investigated formulae and AL, WTW, tKmean values, ELP, decentration and tilt calculated by univariable regression models

For multiple testing, adjusted *P* value (using Bonferroni correction) below 0.0083 indicates that the slope deviates significantly from zero

Values in bold letters represent statistical significance

In a multivariable analysis, the correlation between AL and ELP to the PE using the SRK/T formula in the YG were statistically significant. The Holladay 1 and Hoffer Q formulae of both groups (YG and CG) showed a statistically significant correlation between the ELP and the PE only. The adjusted R^2^ values of the univariable models in the CG group were higher as compared to the R^2^ values of all other uni- and multivariable analyses. A detailed overview can be seen in Table 5.

**Table 5:** Correlation between APE and biometric parameters (multivariable analysis)

Formula		Yamane				Carlevale			
		Estimate	*P* value	VIF	Adjusted R^2^ of model	Estimate	*P* value	VIF	Adjusted R^2^ of model
SRK/T	Intercept	− 4.99	0.247		0.243	− 4.02	0.466		0.408
	AL	− **0.25**	**0.012**	**1.341**		− 0.05	0.556	1.312	
	WTW	0.21	0.431	1.146		− 0.67	0.158	1.774	
	Kmean	0.63	0.106	1.290		0.90	0.183	1.483	
	ELP	**0.82**	**0.037**	**1.382**		**1.31**	**< 0.001**	**1.557**	
	Decentration	− 0.25	0.473	1.226		− 0.70	0.337	1.431	
	Tilt	0.04	0.208	1.075		0.05	0.560	1.771	
Holladay 1	Intercept	− 2.33	0.580		0.095	− 2.34	0.662		0.346
	AL	− 0.15	0.103	1.341		0.02	0.758	1.312	
	WTW	0.12	0.666	1.146		− 0.60	0.194	1.774	
	Kmean	0.16	0.660	1.290		0.41	0.530	1.483	
	ELP	**0.80**	**0.038**	**1.382**		**1.26**	**< 0.001**	**1.557**	
	Decentration	− 0.29	0.412	1.226		− 0.69	0.334	1.431	
	Tilt	0.04	0.214	1.075		0.04	0.647	1.771	
Hoffer Q	Intercept	3.61	0.407		0.255	− 0.82	0.886		0.288
	AL	− 0.17	0.084	1.341		0.02	0.841	1.312	
	WTW	0.10	0.723	1.146		− 0.46	0.342	1.774	
	Kmean	− 0.50	0.203	1.290		0.08	0.905	1.483	
	ELP	**0.80**	**0.043**	**1.382**		**1.20**	**0.003**	**1.557**	
	Decentration	− 0.23	0.516	1.226		− 0.64	0.395	1.431	
	Tilt	0.04	0.276	1.075		0.02	0.813	1.771	

*APE* = arithmetic prediction error; *VIF* = variance inflation factor; *AL* = axial length; *WTW* = white to white; *Kmean* = mean keratometry; *ELP* = effective lens position

Table shows the correlation between the APE of the investigated formulae and AL, WTW, the Kmean values, ELP, decentration and tilt calculated by multivariable regression models.

A *P* value less than 0.05 indicates that the slope deviates significantly from zero

A VIF value less than 4 indicates low collinearity

Values in bold letters represent statistically significance

We did not find any correlation between AL and the ELP (*P* = 0.214, R^2^ = 0.063) nor between WTW and ELP (*P* = 0.715, R^2^ = 0.0062) using univariable regression analyses.

## Discussion

Scleral intraocular lens fixation is gaining more importance over the last years. Subluxated IOLs, pseudoexfoliation syndrome and trauma are the main reasons for scleral IOL fixation. Besides well-established iris fixation and IOL suturing, two relatively new techniques have been developed in recent years. Little is known about the effective lens position, and thus IOL power calculation in these cases is challenging.

The Carlevale and the Yamane methods are two innovative procedures which allow for scleral lens fixation regardless of ACD or iris status. Both techniques are based on the implantation of foldable lenses, which are inserted through a relative astigmatism neutral 2.4–2.8 mm posterior limbal incision, leading to functionally satisfying postoperative results. However, it has to be evaluated if these techniques also fulfill the patient’s increasing expectation of visual function and refractive outcome [9, 10, 13].

Only few studies have reported the refractive outcome of either of the two methods, but so far, none have compared them directly in a prospective manner [11, 14].

Here, we evaluated and compared the PE of 53 consecutive patients who underwent secondary IOL implantation performing the Yamane three-piece IOL technique or secondary Carlevale IOL implantation. Additionally, we analyzed the influence of biometric parameters on the PE.

There was no significant difference in age, postoperative SE, postoperative visual acuity, AL, Kmean, ELP and WTW between the YC and CG.

Surprisingly, there was no statistical difference in IOL decentration (YG: 0.57 ± 0.37 mm, CG: 0.38 ± 0.21 mm) since we expected a higher degree of decentration within the YG as IOL centration is based on the manual adjustment of the IOL position by pulling on the haptics and different flange lengths which appears to be less precise then using the Carlevale IOL.

IOL tilt can cause higher order aberrations and refractive (spherical and torical) errors [15, 16]. Haptic design is very likely responsible for the lower amount of tilt in the CG (6.45 ± 2.03°) as compared to the YG (7.67 ± 3.70°). With the Carlevale IOL, the haptics are placed at the exact opposite positions decreasing the possibility of tilt and decentration. The scleral fixation of a three-piece IOL in the Yamane technique induces torque and stress on the haptic-optic junctions, subsequently leading to IOL tilt. Another source of tilt is that the haptics are passed through an intrascleral tunnel whereby the length and angle of the tunnel in the eye are prone to be asymmetrical despite the external placement of the 30G needle 180° apart. A recent study including 39 patients who underwent Yamane technique reported a mean tilt of 2.4° which was far below our results. But the group measured tilt by calculating the mean of the tilt along the vertical and horizontal axes using an AS-OCT whereas we reported the highest tilt independently of its axis measured over six sectorial B-scans in an AS-OCT [17].

Regarding the calculation of the PE in the YG, we found that the SRK/T formula led to a more myopic result, whereas the Holladay 1 and Hoffer Q to a more hyperopic result than predicted. Our findings considering the Hoffer Q and Holladay 1 formulae are comparable to what was recently published by McMillin et al. [14]. In their study, 40 patients underwent IOL implantation using the Yamane technique with the following formulae being used: Holladay 1, SRK/T Hoffer Q and Barrett formula. Their PEs were between + 0.46 D (Barrett2) and + 0.67 D (Hoffer Q). The difference with the SRK/T results between their hyperopic shift and our myopic shift is somehow surprising, as they stated that their haptic externalization points were placed 2 mm behind the limbus as originally described by Yamane [8], whereas we chose 2.5 mm so we would expect a hyperopic shift. This incongruence might be due to forward or backward shift of the IOL optic compared to the haptic fixation point or high variability between the surgeons’ personal definition of the surgical limbus as a landmark. Due to some cases of optic-iris-capture within our pilot series, which we performed prior to this study, we chose to externalize the haptics 0.5 mm posterior to the recommended location. The lower rates of optic-iris-capture in Yamane’s patients, in the above-mentioned study, might be due to the use of an IOL with a 7 mm optic diameter, compared to the IOL we implanted with a common optic diameter of 6 mm [8].

The evaluation of the Barrett formula is of subordinate importance as it is based on ACD and lens thickness, of which both are not available in pseudophakic or aphakic patients. Considering the MAE, they reported values between 0.73 D (Holladay 1) and 0.86 D (Hoffer Q), which are comparable to ours [14].

Compared to the Yamane method, the IOL haptics in the Carlevale method are externalized 1.5 mm posterior to the limbus. However, this IOL is specifically designed for scleral fixation with a particular optic and haptic design. The optic diameter is 6.5 mm and each haptic is attached at two points on the optic converging into one anchor haptic creating a kind of plate haptic with a single scleral attachment on each side of the optic. We did not observe any optic-iris-capture in our study patients. The IOL power constants are adjusted to this scleral fixation point, and thus in the CG, we found that SRK/T and Holladay 1 formula resulted in a more myopic outcome than predicted. On the contrary, the Hoffer Q resulted in a more hyperopic outcome, similar to that in the YG.

Our PE results in the CG agree with the ones published by Vaiano et al. [11]. They retrospectively analyzed the refractive PE of 25 patients and adapted the IOL power calculation constants to subsequently level the mean PE to zero. They reported slightly higher standard deviations for the PEs which were between ± 0.89 D (SRK/T) and ± 0.95 D (Hoffer Q) depending on the formula used, compared to ours which were between ± 0.74 D (Holladay 1) and ± 0.80 D (SRK/T).

They reported MAEs between 0.62 D (SRK/T) and 0.67 D (Hoffer Q) which were similar to our results 0.61 D (Holladay 1) and 0.68 D (SRK/T).

The proportion of eyes within ± 0.5 D was higher in Vaiano’s analysis (56% SRK/T to 64% Holladay 1) compared to ours (45% SRK/T to 52% Holladay 1), though in our study, more eyes were within ± 1.0 D (68% Holladay 1, 72% Hoffer Q & SRK/T in Vaiano et al.’s study; 72% SRK/T, 76% Hoffer Q & Holladay 1 in our study) and none were beyond 2.0 D [11].

Across our groups, no difference in the PE between corresponding formulae could be found. Concerning the AE, no difference between the formulae could be observed within and across groups.

Multiple studies reporting the PE after Iris claw implantation showed a PE between 0.99 ± 0.57 D and 1.1 ± 0.94 D. The proportion of eyes within ± 1.0 D AE was around 60%. The PE and AE using the Yamane or Carlevale method were lower as compared to the above results [18–20]. Besides inferior refractive outcome, iris claw lenses need larger incisions which result in higher postoperative astigmatism and possible wound leakage in the early postoperative follow-up [18, 21, 22].

Modern formulae lead to a highly predictable refractive outcome after cataract surgery in which 66%–71% of eyes are within an error range of 0.5 D and between 90% and 97% within 1.0 D depending on eye length, formula and publication [23–25]. Scleral fixated IOLs are not as precise as compared to in-the-bag IOL implantation. Possible reasons are effects of biometric parameters on the power calculation and high variabilities in the ELP. Additionally, patient numbers are much lower and subsequently, extreme eye lengths are rarer and therefore statistical analyses and IOL constant optimization become less precise.

Regarding the influence of presurgical biometric data on the PE, we did not find any statistically significant nor clinically relevant correlations between AL, Kmean and the PE in the CG, so no recommendation about which formula to use in certain eye lengths and corneal radii can be made.

We did not find any significant correlation between AL and the PE in the YG which is in line with what was published by McMillin [14]. Although we did not find any correlation between WTW and the PE in either group, Mularoni et al. reported that two of their patients with an WTW of over 12.6 mm led to extensive haptic stretching which shifted the FIL-SFF IOL optic anteriorly [26]. Three of our patients showed a WTW above 12.5 mm, however we only observed a slight myopic shift in one of the patients (Holladay 1: −0.5 D). Furthermore, we did not find any significant correlations between AL and ELP nor WTW and ELP.

Our final goal was to evaluate the influence of the ELP on the PE. In the CG, increasing ELP led to a more hyperopic result in both uni- and multivariable analyses, reaching R^2^ values from 0.28 up to 0.44 whereas there was only very little correlation (max R^2^ = 0.2) between ELP and PE for the YG. This discrepancy might be explained by differences in tilt or the fewer number of patients.

For the CG however, ELP was the parameter with highest influence on refractive outcome in our analysis, highlighting the importance of reliable predictability of the IOL position and reliable intraoperative lens positioning. Similarly, a clinically relevant correlation between ELP and PE was found for sutured secondary IOL fixation after pars plana vitrectomy (ppVE), where a 1.0 mm more posterior externalization point led to a + 1.0 D hyperopic PE [27]. Hence, for scleral lens fixation techniques, precise and reliable placement of haptics is an important factor for ELP and the final refractive outcome, especially when compared to in-the-bag IOL placement, where the ELP is less likely to be influenced directly by the surgeon.

The results of our study must be interpreted with certain limitations as the number of patients in both groups is small and there are no eyes with very long or very short AL or large WTW values. Although small astigmatism-neutral posterior limbal incisions were made, minimal amounts of corneal astigmatism could be induced and subsequently influence the refractive outcome. Additionally, while all surgeries were performed by a single, highly experienced surgeon, scleral IOL fixation may be affected to a higher degree by individual operating style and technique in comparison to primary in-the-bag placement.

## Conclusions

We show that both, the Yamane and Carlevale techniques, result in good predictable outcomes. In our cohort, the PE was closest to zero using the Holladay 1 formula, although there was no statistically significant difference to the other formulae. ELP was the most relevant factor for PE, especially in the CG, underscoring the importance of precise haptic externalization placement. However, further research is necessary to better understand the correlations between surgical anatomical landmarks such as the limbus, and AS-OCT landmarks such as the scleral spur to subsequently better predict the ELP for the implantation of scleral fixated lenses. This would ultimately lead to better refractive results especially in eyes, with very high or low of AL and WTW measurements.

## Data Availability

The datasets generated during and/or analyzed in this study are not publicly available due to privacy regulations set by the Medical University of Vienna but are available from the corresponding author on reasonable request.
